# Cellular Reprogramming and Its Potential Application in Alzheimer’s Disease

**DOI:** 10.3389/fnins.2022.884667

**Published:** 2022-04-07

**Authors:** Chao Zhou, Wanyan Ni, Taiyang Zhu, Shuyu Dong, Ping Sun, Fang Hua

**Affiliations:** ^1^Institute of Neurological Diseases, Xuzhou Medical University, Xuzhou, China; ^2^Department of Neurology, The Affiliated Hospital of Xuzhou Medical University, Xuzhou, China; ^3^Department of Neurology, Xuzhou Central Hospital, Xuzhou, China; ^4^Department of Neurology, School of Medicine, University of Pittsburgh, Pittsburgh, PA, United States

**Keywords:** Alzheimer’s disease, cellular reprogramming, iPSCs, neuroregeneration, neurological function

## Abstract

Alzheimer’s disease (AD) has become the most common age-related dementia in the world and is currently incurable. Although many efforts have been made, the underlying mechanisms of AD remain unclear. Extracellular amyloid-beta deposition, intracellular tau hyperphosphorylation, neuronal death, glial cell activation, white matter damage, blood–brain barrier disruption, and other mechanisms all take part in this complicated disease, making it difficult to find an effective therapy. In the study of therapeutic methods, how to restore functional neurons and integrate myelin becomes the main point. In recent years, with the improvement and maturity of induced pluripotent stem cell technology and direct cell reprogramming technology, it has become possible to induce non-neuronal cells, such as fibroblasts or glial cells, directly into neuronal cells *in vitro* and *in vivo*. Remarkably, the induced neurons are functional and capable of entering the local neural net. These encouraging results provide a potential new approach for AD therapy. In this review, we summarized the characteristics of AD, the reprogramming technique, and the current research on the application of cellular reprogramming in AD. The existing problems regarding cellular reprogramming and its therapeutic potential for AD were also reviewed.

## Introduction

Currently, Alzheimer’s disease (AD) is the most common cause of dementia. Due to the continuous improvement in living standards and the aging of the population, the prevalence of AD is gradually increasing. According to the Alzheimer’s Association, an estimated 6.2 million Americans aged 65 and older currently live with Alzheimer’s dementia, and this number could grow to 13.8 million by 2060, barring the development of medical breakthroughs to prevent, slow, or cure AD (Alzheimer’s Association, 2021). The death rate due to AD and other dementias throughout the world increased by 38.2% (Scheltens et al., 2016). AD has critically affected the quality of life of the elderly, and this trend will continue in the next few decades. However, so far, there has been no effective treatment for this disease.

Reprogramming refers to the erasure and remodeling of epigenetic marks. Cellular reprogramming technology is a traditional but developing method sequenced by Gurdon’s pioneering experiments on nuclei implantation to convert cell differentiation fate (Gurdon, 1962). In a previous study, four transcription factors—Oct4, Sox2, Klf4, and c-Myc—were delivered to mouse fibroblasts by applying retrovirus, miraculously reversing the cells’ fate to a pluripotent state, which were called induced pluripotent stem cells (iPSCs) (Takahashi and Yamanaka, 2006). Reprogramming technology enables differentiated cells of a specific cell type to be converted to another cell type with completely different functions, either through the production of iPSCs or through direct conversion. This technique has been utilized to establish AD-derived models from different cells, including mononuclear cells from peripheral blood, hair follicles, skin fibroblasts, exfoliated renal epithelial cells, etc. (Takahashi and Yamanaka, 2006; Zhou et al., 2012; Hoffman, 2016; Lee et al., 2016; Uhm et al., 2017). Amyloid-beta (Aβ) extracellular deposits and tau protein hyperphosphorylation were investigated using AD-derived models (Fujiwara et al., 2015; Nieweg et al., 2015; Brownjohn et al., 2017; Cahill and Huang, 2017).

*In vivo* cellular reprogramming is another momentous technology co-developed with iPSC technology. *In vivo* reprogramming uses transdifferentiation, in which the source cell type is directly transformed into the target cell type without intermediate pluripotent cells or stem cell stages, directly affecting the transformation of cells *in vivo* (Srivastava and DeWitt, 2016). *In vivo* cellular reprogramming has been achieved in the liver, heart, and pancreas (Zhou et al., 2008; Banga et al., 2012; Qian et al., 2012), which motivates the evolution of *in vivo* reprogramming in the brain (Niu et al., 2013; Guo et al., 2014). Compared to iPSCs, *in vivo* reprogramming is a unique approach in that cell culture and subsequent engraftment under variable *in vitro* incubator conditions are not necessary. Research on *in vivo* reprogramming methods includes *in situ* generation or regeneration of cardiomyocytes, pancreatic β cells, and neurons for regenerative medicine, and regeneration/anti-aging (Torper et al., 2013; Cavelti-Weder et al., 2016; Gong et al., 2021). Recently, a growing number of experimental studies have reported the design of *in vivo* programming to treat AD and reduce its pathological changes and neurological dysfunction (Guo et al., 2014; Hu et al., 2015; Baik et al., 2019; Rodriguez-Matellan et al., 2020). In this review, we briefly summarize the current state of AD and the application of *in vivo* reprogramming in AD.

## Etiology of Alzheimer’s Disease

Alzheimer’s disease is a progressive retrograde illness characterized by progressive cognitive impairment and behavioral dysfunction. At the terminal stage, the patient cannot complete daily activities, such as dressing and eating. Based on the age of onset, AD can be classified as early-onset AD (EOAD) or late-onset AD (LOAD). The signs and symptoms of EOAD appear between ages 30 and 60 (Long and Holtzman, 2019), whereas LOAD appears during or after age 60. EOAD is much less common than LOAD, and it accounts for less than 10% of all AD cases. Early-onset familial AD is inherited in an autosomal dominant pattern and is usually associated with the amyloid precursor protein (APP), presenilin 1 (PSEN1), and presenilin 2 (PSEN2) gene mutations (Kent et al., 2020). APP protein, encoded by APP gene, is proteolytically processed by β- and γ-secretase into Aβ peptides of various lengths. Most pathogenic mutations in APP have been reported to either increase Aβ production or affect the ratio of Aβ peptides of different lengths, such as Aβ42/Aβ40 ratio, leading to increased self-aggregation (Weggen and Beher, 2012). The PSEN1 gene provides instructions for making PSEN1 protein, a subunit of γ-secretase complex. The γ-secretase complex cuts APP into smaller peptides, including soluble amyloid precursor protein (sAPP) and several versions of Aβ peptide. PSEN1 gene mutations have been identified in patients with EOAD, accounting for up to 70% of the cases. These mutations lead to the production of an abnormal PSEN1, impair the function of the γ-secretase complex, alter the processing of APP, and result in the overproduction of a longer, toxic version of Aβ (Sun et al., 2017). The function of PSEN2 is to help process proteins that carry chemical signals from the cell membrane to the nucleus. PSEN2 works with other enzymes to cleave APP into smaller fragments (peptides). The mutations in PSEN2 gene appear to disrupt the processing of APP, resulting in an overproduction of Aβ peptide (Cai et al., 2015). EOAD cases account for 5–10% of AD cases, but only 10–15% of these cases show known mutations in APP, PSEN1, and PSEN2 associated with EOAD (Ayodele et al., 2021), which suggest that only a small fraction of this genes with mutations has been identified, and there is still a lot of work to be done to discover additional disease-causing genes.

The inheritance pattern of LOAD is uncertain. However, apolipoprotein E (APOE) has been found to be associated with the pathogenesis of LOAD. The APOE gene encodes apolipoprotein E, which combines with lipids to form lipoproteins and is involved in maintaining normal levels of cholesterol. The APOE gene has at least three different alleles, called e2, e3, and e4. People who inherit one copy of the APOE e4 allele have an increased chance of developing LOAD. Those who inherit two copies of the allele are at even greater risk of developing LOAD. The mechanism by which the APOE allele increases the risk of LOAD is unclear, but it may be related to their pleiotropic functions that lead to reduced cholesterol transport, less efficient Aβ clearance and more aggregation, and triggering neurotoxicity through Tau phosphorylation (Shi et al., 2017; Lewandowski et al., 2020). In addition to possible genetic factors, a variety of acquired factors can lead to the development of AD. Aging or cellular senescence is believed to contribute importantly to aging and aging-related diseases, including LOAD (Liu, 2022). Early life stress and environmental neurotoxic may be risk factors for the initiation and progression of LOAD (Gauvrit et al., 2022; Tsamou et al., 2022). The GG and AG genotypes of the insulin-degrading enzyme (IDE) gene SNP rs2421943 may affect the rate of IDE pre-RNA (heterogeneous nuclear RNA, hnRNA) processing, resulting in slower translation, reduced IDE levels, insufficient removal of Aβ fragments, and increased risk and/or accelerated progression of AD (Šerý et al., 2022).

In general, the etiology of AD remains unclear. Early-onset familial AD is closely related to genetic factors, while for late-onset sporadic AD, in addition to genetic factors, a variety of acquired factors are related to its onset and progression, such as aging, inflammatory response, traumatic brain injury, ischemia, diabetes, and toxic. More detailed information has been reviewed (Rabinovici, 2019). The existing research supports the notion that AD is a heterogeneous disease involving multiple pathogenic factors (Lin et al., 2020; Nardini et al., 2021).

## Main Molecular Factors Involved in the Pathogenesis of Alzheimer’s Disease

The APP is an integral membrane protein with a single membrane-spanning domain, an extracellular N-terminus, and a cytoplasmic C-terminus (Kent et al., 2020). APP is proteolytically hydrolyzed into different forms of Aβ fragments by β-secretase and γ-secretase, and further cleaved into 40-amino acid Aβ40 and 42-amino acid Aβ42. There are three main types of Aβ peptides: Aβ1–40, Aβ1–42, and Aβ1–43 (Selkoe and Hardy, 2016). The C-terminus of Aβ42 is less flexible than that of Aβ40 and contributes to the main sources of amyloids (Chen et al., 2017). Aβ42 and Aβ43 are β-sheet structures with strong hydrophobicity, easy deposition, and neurotoxicity. Under normal circumstances, Aβ40 accounts for 90%, with only a small amount of Aβ42 and Aβ43. In the brains of AD patients, Aβ42/43 is elevated, resulting in imbalance of their ratio to Aβ40 (Selkoe and Hardy, 2016). Soluble Aβ oligomers can spread throughout the brain and cause extensive neurotoxicity. Conversely, fibrillar Aβ are insoluble, and they can be assembled and localized to form senile plaques (Chen et al., 2017). On the other hand, cerebral aggregated Aβ can be degraded by several processes, including proteolytic degradation, cell-mediated clearance, active transportation, and deposition into insoluble aggregates (Hillen, 2019). Neprilysin, endothelin-converting enzymes 1 and 2, insulin-degrading enzyme, plasmin, and other Aβ-degrading proteases are involved in this process (Miners et al., 2008; Hillen, 2019). In addition to the degradation process, Aβ can also be transported to the extracellular space and out of the brain. Intracellular and extracellular Aβ can also activate microglia to uptake Aβ through immune-inflammatory regulatory pathways. However, the hyperactivated microglia can induce neuron loss through phagocytosis (Liu et al., 2012; McDonald et al., 2016). For example, Toll-like receptors in microglia are essential for the process of Aβ uptake, however, their activation also induce chronic inflammation, which accelerates the progression of AD (Stewart et al., 2010).

PSEN1 and PSEN2 are presenilin proteins encoded by the PSEN1 and PSEN2 genes in humans. Nearly all variants of the PSEN genes alter single DNA (nucleotide) building block in a specific segment of the PSEN genes. As mentioned before, these variants lead to the production of abnormal presenilin proteins, which interfere with the function of the γ-secretase complex, altering APP processing and leading to overproduction of the longer, toxic Aβ peptide (Cai et al., 2015; Sun et al., 2017).

The APOE is highly expressed in astrocytes and microglia to mediate the uptake and transportation of Aβ. APOE has three genetic isoforms: APOE2, APOE3, and APOE4. The APOE4 isoform affects the production, clearance, and/or toxicity of Aβ, and is the most important genetic risk factor for AD, whereas APOE2 reduces AD risk (Huang et al., 2017). APOE is the gene most associated with AD among other identified candidate genes, including the ATP binding cassette subfamily A member 7, bridging integrator 1, CD33 molecule, clusterin, complement C3b/C4b receptor 1, CD2-associated protein, ephrin type-A receptor 1, membrane-spanning 4-domains A6A-A4E, phosphatidylinositol-binding clathrin assembly protein, HLA class II histocompatibility antigen, DRB5 beta chain- DRB1 beta chain, sortilin-related receptor 1, fermitin family homolog 2, Cas scaffold protein family member 4, and protein tyrosine kinase 2 beta (Scheltens et al., 2016).

Tau proteins are a group of six highly soluble protein isoforms produced by alternative splicing of the gene MAPT (microtubule-associated protein tau). They play a major role in maintaining the stability of microtubules in axons, and are abundant in neurons, but at very low levels in astrocytes and oligodendrocytes, in central nervous system (Amir Mishan et al., 2019). As an important cellular structure, microtubules play a crucial role in maintaining cell morphology, generating cell polarity, and transporting intracellular substances (Muralidar et al., 2020). The hyperphosphorylation of tau leads to the destabilization of microtubules, which eventually leads to the disassembly of microtubules and the misfolding of tau proteins to form paired helix-like dimers that are neurotoxic and ultimately cause neuronal degradation (Chong et al., 2018). Excessive or abnormally phosphorylated tau proteins lose their role in maintaining the stability of microtubules, causing microtubule depolymerization, axonal transport dysfunction, and synaptic dysfunction, which in turn leads to neuron degeneration and neuronal apoptosis, signaling the occurrence of AD (Muralidar et al., 2020).

## Pathological Features of Alzheimer’s Disease

The first core pathological feature of AD is Aβ plaques. Aβ plaques are composed of Aβ containing 41–43 amino acids deposited outside the cell to form the core, surrounded by degenerated axons, dendrites, amyloid fibers, glial cell protrusions, and a crown formed by microglia (Graff-Radford et al., 2021). The Aβ cascade hypothesis considers that the deposition of Aβ in the brain is the central link of AD pathological changes; it triggers a series of pathological processes, further promotes the deposition of Aβ, and forms a cascade amplification reaction. The increased deposition of Aβ42/43 forms the core of senile plaques, which in turn activates microglia and triggers an inflammatory response, leading to mitochondrial damage, energy metabolism disorder, oxidative stress damage, and activation of the apoptosis pathway. In addition, Aβ42/43 can damage cholinergic neurons and cause lesions in the acetylcholine system. Aβ42/43 can also form oligomers of APP and mediate the neurotoxicity of Aβ (Murakami et al., 2016; Hillen, 2019).

The second core pathological change is Neurofibrillary tangles (NFTs), which are located in the neuronal cytoplasm. Their main component is the aberrantly phosphorylated microtubule-associated tau protein (Bennett et al., 2017; Fu et al., 2017). Normal tau proteins are microtubule-associated proteins that maintain the stability of the cytoskeleton by binding to microtubules. The hypothesis of abnormal phosphorylation of tau proteins states that the inflammatory response in AD brain tissue can activate protein kinases, promote the abnormal phosphorylation of tau proteins, and make them aggregate to form double-stranded helical filaments, which form NFTs, resulting in neurotoxicity. Along with the reduction of normal tau proteins, microtubules collapse and axoplasmic transport is interrupted or disturbed, resulting in axonal degeneration, neuronal mitophagy (Martinez-Vicente, 2017), and neuronal death (Gupta and Goyal, 2016; Schöll et al., 2016).

The third core pathological feature of AD is a persistent immune/inflammatory response, which was identified in the previous decade. It not only provides an in-depth understanding of AD pathogenesis but also a link between the previous two core pathologies. Recent studies have shown that the disruption of the balance between anti-inflammatory and pro-inflammatory signals in the brain tissue of AD patients leads to chronic neuroinflammation, which is associated with activated microglia and the release of various cytokines (Sonninen et al., 2020; Yang and Zhang, 2020). Persistent immune/inflammatory responses in the brain are not only associated with neuronal loss and neurodegeneration but are also closely related to the initial Aβ pathology and its interaction with NFTs (Otani and Shichita, 2020). The pathological changes in Aβ and tau in the brain cause excessive activation of astrocytes and microglia, express a large number of inflammatory substances, and produce an excessively sustained chronic inflammatory response, which leads to neuronal dysfunction, degeneration, and apoptosis (Calsolaro and Edison, 2016; Zhou et al., 2019). Unlike genetic causes and other risk factors, neuroinflammation is generally not considered to be the initial cause of AD but is more likely to be the result of one or more AD pathologies or associated risk factors, which increase AD severity by exacerbating the pathological processes of Aβ, tau proteins, and NFTs (Kinney et al., 2018; Paouri and Georgopoulos, 2019; Yang and Zhang, 2020). The common pathway of neuronal death in neurodegenerative diseases, including AD, is caused by oxidative stress, dysregulated calcium homeostasis, excess production of free radicals, and calcium overload, causing the disruption of mitochondrial membranes and mitochondrial dysfunction (Egawa et al., 2020). While neurogenesis is a highly energy-intensive process, mitochondrial dysfunction can easily lead to neuronal death (Rudnitskaya et al., 2022).

## Therapeutic Approaches for Alzheimer’s Disease

The pathological changes in AD are diverse and complex, and its pathogenesis remains unclear. Thus far, there have been no drugs or other interventions that can effectively cure the disease. Currently, effective nursing from family and others is paramount to the treatment of AD. Traditional drug treatment (e.g., donepezil, rivastigmine, galantamine, and memantine) is also generally used to alleviate symptoms temporarily (Canter et al., 2016). However, solanezumab, a drug based on the amyloid hypothesis, has failed in multiple trials (Abbott and Dolgin, 2016). Even worse, this is just one of several setbacks (Anderson et al., 2017; Mehta et al., 2017). Although another Aβ-directed antibody, aducanumab, was recently approved by the Food and Drug Administration, the drug’s effect on the surrogate endpoint is expected but not established (Howard and Liu, 2020).

Unlike in other organs, a critical issue to be surmounted in the brain is that it has a blood–brain barrier (BBB). Oral drugs can only partly enter the bloodstream and cannot efficiently enter the encephalon and foci. Considering this issue, Kuo and Lee used serotonin modulators and ApoE-conjugated liposomes, a nanomedicine technique that could build up the nerve growth factor’s BBB permeability, thereby generating neuron anti-apoptosis (Kuo and Lee, 2016). Unfortunately, despite various measures and similar studies, most AD patients die of complications after 5–10 years.

This reality reminds us that traditional medicines are running into bottlenecks and that effective medicines are still pending. Finding a novel way to alleviate cognitive impairment in AD has become extremely urgent. At present, several possible interventions, including immunotherapy, pharmacotherapy, cognitive training, physical exercise, and the treatment of cardiovascular disease and diabetes, are under investigation.

In AD, large numbers of neurons are damaged and die, the connections between neuronal networks are also disrupted. Unfortunately, endogenous neurogenesis and gliogenesis decline significantly with age, failing to regenerate enough brain cells to adequately mitigate AD-induced neurodegeneration. Therefore, transplantation of exogenous stem cells into AD brain to generate new neurons has attracted much attention and been extensively studied. Currently, there are mainly the following types of stem cells used for therapeutic purposes, including embryonic stem cells (ESCs), neural stem cells (NSCs), mesenchymal stem cells (MSCs), olfactory ensheathing cells (OECs), hematopoietic stem cells (HSCs), and induced pluripotent stem cells (iPSCs) (Alipour et al., 2019). The therapeutic concepts are that the transplanted stem cells can migrate to the site of injury (Imitola et al., 2004; Carbajal et al., 2010; Huang et al., 2014), and are able to differentiate into neurons and glial cells to replace dead or dying brain cells. In addition, the stem cells secrete therapeutic gene products, such as neuroprotective growth factors, as well as stimulate endogenous repair mechanisms, and create a favorable microenvironment for host cell survival and normal functioning of the CNS (Lee et al., 2007; Boese et al., 2020). Stem cell therapy has great potential in the treatment of AD, and a growing number of basic research and clinical trials using stem cells to treat AD are still ongoing. Each type of stem cells has its own advantages and disadvantages. Some shortcomings greatly affect the application of stem cells and remain unresolved, for example, ethical issues, uncontrolled differentiation, possible pathological phenotypes, and low neuronal differentiation and survival rates (Vasic et al., 2019). These issues make translation from rodent models to clinical application still difficult, requiring more basic and clinical research. Importantly, other novel approaches may also be an option, including cellular reprogramming.

## Cellular Reprogramming Technology

Reprogramming technology is a method of transforming differentiated cells of a particular cell type into another cell type with a completely different function. In the past, biologists believed that the process of naive cells to maturity was an irreversible process. However, this perception has been changed. Metaplasia and carcinogenesis are, to a certain extent, autologous cell reprogramming under pathological conditions, which, in many cases, are completely uncontrollable and detrimental to our health (Ladewig et al., 2013; Srivastava and DeWitt, 2016). Gurdon established the first artificial reversal of mature somatic cells by integrating the enucleated oocyte with frog somatic cells, finally giving rise to a new organism (Gurdon, 1962). Afterward, Wilmut created Dolly, a cloned sheep, which demonstrated for the first time that cellular reprogramming could be produced in mammals (Wilmut et al., 1997).

Aside from nuclear transplantation, Gehring initiated the use of a single transcription factor (TF) MyoD to convert fibroblasts to myoblasts, thereby conducting direct cellular reprogramming (Davis et al., 1987). In parallel with direct cellular reprogramming, Yamanaka and colleagues created induced pluripotent stem cells (iPSCs), which utilize several TFs to convert somatic cells into pluripotent stem cells. This epoch-making invention has changed previous perspective and promptly applied to various fields of life science. Various germlines, different tissues, and originated cells have been successively induced into iPSCs (Takahashi and Yamanaka, 2006; Zhou et al., 2012; Hoffman, 2016; Lee et al., 2016; Uhm et al., 2017). These findings have been applied to help scientists address neurological diseases. Heins et al. (2002) discovered a master transcriptional regulator that controls the neuron cell fate and reverses glial cells to neurons using Pax6, a single transcriptional factor. Vierbuchen et al. (2010) demonstrated that a combination of three factors, Ascl1, Brn2, and Myt1l, could make a lineage conversion from fibroblasts to functional neurons. Interestingly, fibroblasts can be directly reprogrammed into astrocytes and oligodendrocytes (Caiazzo et al., 2015).

Another approach to reprogram cells is small molecule cocktail without TFs. This method is considered safer than TFs, such as VCR cocktail (V, valproic acid; C, CHIR99021; and R, Repsox), a cocktail of nine small molecules (LDN193189, SB431542, TTNPB, Tzv, CHIR99021, VPA, DAPT, SAG, and Purmo), and the FICS cocktail (Forskolin, ISX9, CHIR99021, and SB431542) (Hu et al., 2015; Li et al., 2015; Zhang et al., 2015).

The iPSCs can be used for cell transplantation therapy due to their pluripotency, which has been used for the first time in humans. In the clinic trial, two neovascular (also called “wet”) age-related macular degeneration patients were recruited to receive autotransplantation. Retinal pigment epithelial (RPE) cells derived from skin fibroblasts–iPCSs (iRPE cells) were strictly detected. Then, one of the patients underwent iRPE cell autografting in 2014. Since then, the step of the patient’s macular degeneration has been stopped. Another patient’s transplant program halted due to DNA aberrations in the iRPE cell process (Mandai et al., 2017). Unfortunately, another clinical trial ended with a patient’s vision loss (Kuriyan et al., 2017).

## Application of *in vitro* Reprogramming in Alzheimer’s Disease

The iPSCs method has been applied to the study of AD etiology and pathogenesis. New pathogenic mechanisms are constantly being revealed. For example, Usenovic et al. (2015) used human neurons derived from iPSCs seeded with full-length human tau monomers and oligomers, and reported that Tau oligomers, not monomers, were responsible for tau accumulation. AD patient iPSC-derived neurons recapitulate the AD phenotypes of amyloid aggregation, hyperphosphorylated tau protein, and endosome abnormalities (Raja et al., 2016). Moreover, astrocytes derived from AD-iPSCs, also reconstruct an abnormal morphology and dysfunction of maintaining a neuron network (Jones et al., 2017).

Cellular models play an important role in understanding the pathogenesis of sporadic AD. Cellular reprogramming and epigenetic techniques provide new avenues for modeling this disease. Liu and Wang (2020) reported that fibroblasts were first infected with a retrovirus overexpressing human SOX2, and 12 days later, cells were treated with 9 small molecule compounds (M9: CHIR99021; A83-01; RG108; Parnate; SMER28; Hh-Ag 1.5; LDN193189; retinoic acid; and bFGF) for an additional 6–8 days until typical stem cell colonies appear. Using this combined approach, AD and wild-type (WT) iNSC lines were generated from primary fibroblasts (Liu and Wang, 2020). These cells possessed the typical neural stem cell properties and were able to be further differentiated into neurons and glia *in vitro* and *in vivo*. More importantly, the AD iNSC derived neurons replicated the major neuropathological features of AD, indicating its role as a useful tool for studying sporadic AD pathogenesis and drug discovery (Liu and Wang, 2020). Interestingly, when neurons derived from human-induced pluripotent stem cells (hiPSCs) were grafted into a mouse model of AD, hiPSCs-neurons exhibit features of neurodegeneration (Espuny-Camacho et al., 2017). Further, AD-iPSC-derived neural progenitor cells (which carry a PSEN1 gene mutation) showed increased apoptosis and decreased proliferation (Yang et al., 2017), and hiPSC-derived astrocytes (which carry an APOE ε4 gene mutation) exhibited a diminished neurotrophic function (Zhao et al., 2017). In turn, APOE ε4 accelerated the production of Aβ and APP (Huang et al., 2017). Moreover, protein-iPSCs grafted to a 5xFAD transgenic AD mouse model reduced plaque deposition and relieved cognitive dysfunction (Cha et al., 2017).

In the initial experimental stage, exogenous transcription factors increased the likelihood of oncogenicity, and the reprogramming-generated iPSCs were less efficient than expected. After more than 10 years of research, these problems have been initially solved. The efficiency of reprogramming could be enhanced by using antioxidants, an anti-apoptotic protein, the brain-derived neurotrophic factor, and other substances (Gascón et al., 2016; Chung et al., 2017; Mattiassi et al., 2021). Safety concerns have been addressed using non-integrating vectors, including Sendai viral, episomal, and mRNA (Schlaeger et al., 2015). A similar breakthrough has occurred in direct cell reprogramming *in vitro*. However, similar to iPSCs, *in vitro* direct reprogramming also requires a grafting process for use in animal models or patients, which may pose a secondary injury to brain. In addition, iPSC implantation technique experiences a long waiting time for acquiring a sufficient yield of patient-specific iPSCs and iPSC-induced neural precursor/stem cells or neurons. In the future, more research should be done on iPSC allografts, xenografts, and the cell types best suited for reprogramming.

## *In vivo* Cellular Reprogramming for Brain Repair

Recently, there has also been a breakthrough in a type of direct cell reprogramming called *in vivo* cell reprogramming, which is a therapeutic approach to regenerative medicine aimed at delivering a cell-based therapy to an organ or tissue in need of functional restoration without the use of a cellular agent (Srivastava and DeWitt, 2016). For example, in adult pancreas, three factors were used to convert exocrine cells of differentiated pancreatic cells into β-cells, a type of insulin^+^ cells that can release insulin (Zhou et al., 2008). In the liver and heart, several TFs have been proven to achieve *in vivo* cellular reprogramming (Banga et al., 2012; Qian et al., 2012). In a research on neuroregeneration, post-mitotic corpus callosum neurons were directly converted into corticofugal neurons following expression of the transcription factor encoded by Fezf2 *in vivo* (Rouaux and Arlotta, 2013). Furthermore, by utilizing a single TF Sox2, resident astrocytes are sufficient to surmount a lineage restriction to a neuroblast state, and they can continuously exist in the mouse brain (Niu et al., 2013). Currently, polypyrimidine tract-binding protein 1 (Ptbp1) has attracted the attention of researchers due to its properties in central nervous system (CNS) reprogramming. Inhibition of Ptbp1 gene expression in a Parkinson’s disease mouse model induces astrocytes to dopaminergic neurons (Qian et al., 2020). With the CRISPER/CasRx technology, astrocytes can be converted into dopaminergic neurons by downregulating the Ptbp1 gene (Zhou et al., 2020). Ptbp1 downregulation also converted the established central projections of retinal ganglion cells (RGCs) into dorsal lateral geniculate nucleus and superior colliculus in an NMDA-induced retinal injury mouse model (Zhou et al., 2020). Through the intracranial injection of NeuroD1 and Dlx2 rAAV2/5, astrocytes are converted into GABAergic neurons in a Huntington’s disease mouse model (Wu et al., 2020). Moreover, reactive astrocytes can be converted by SOX2 into oligodendrocyte lineage cells in adult demyelinated brain (Farhangi et al., 2019). Non-reactive astrocytes can undergo neuronal conversion by delivering NeuroD1 via the AAV9 intravascular path (Brulet et al., 2017). In addition to TF-mediated and Ptbp1 knockdown *in vivo* reprogramming, astrocytes attain transdifferentiation through the miR-302/367, a microRNA-mediated path (Ghasemi-Kasman et al., 2015). Compared with previously used viral vectors, these new findings make *in vivo* reprogramming therapeutics safer and more reliable. The recent novel findings of *in vivo* reprogramming strategies to treat neurological diseases are listed in Table 1.

**TABLE 1:** *In vivo* cellular reprogramming in neurological system.

Model	Reprogramming factors	Carrier	Region	Type of conversed cell	Type of converted cell	Functional analysis of converted cells	Outcomes	Route	References
Intact mouse brain	Chemical cocktail (Forskolin, ISX9, CHIR99021, and I-BET151)	N/A	Intact striatum and cortex	Resident astrocytes	GABAergic neurons (DARPP32+, NPY+, and PVALB+). Cortex-specific neurons (CTIP2+, TBR1+, and PVALB+)	Chemically induced neurons showed the ability to receive synaptic projections from the host neurons.	N/A*	Intracranial injection (Alzet osmotic minipumps)	Ma et al., 2021
Mouse model of middle cerebral artery occlusion	NeuroD1	Lentivirus	Peri-infarct region	Reactive astrocytes	Glutamatergic neurons (vGLUTs+)	Induced neurons increased BDNF, FGF10, and PSD-95 expression, whereas reduced inflammatory protein expression (NF-κB, Iba-1) in peri-infarct regions.	Astrocyte reprogramming improves sensorimotor functional outcomes after stroke	Intracranial injection	Jiang et al., 2021
Rat model of traumatic spinal cord injury	Recombinant Neuregulin-1	N/A	Injured spinal cord	Reactive astrocytes	Oligodendrocytes (PDGFRα+, O4+, and CNPase+)	N/A	Converted oligodendrocytes inhibited astrogliosis, promoted remyelination, protected axons and eventually improved BBB score	Intrathecal delivery (Alzet osmotic pumps)	Ding et al., 2021
Wide type mouse. Huntington’s disease (R6/2 mouse model)	NeuroD1 and Dlx2	rAAV2/5	Intact striatum. R6/2 mouse striatum	Astrocytes	GABAergic neurons. (NeuN+, GAD67+, GABA+, DARPP32+, PV+)	Converted neurons showed similar amplitude and typical MSN firing pattern to the WT neurons and can be incorporated in local synaptic circuits	*In vivo* regeneration of GABAergic neurons in the striatum of R6/2 mice can partially rescue the phenotypic deficits and extend the life span	Intracranial injection	Wu et al., 2020
Wild type mouse. 6-OHDA induced Parkinson’s disease (PD) mouse model	PTB (Ptbp1) shRNA. PTB antisense oligonucleotides (PTB ASOs)	AAV2	Intact midbrain. PD midbrain	Astrocytes	Mature neurons (NeuN+, MAP2+, NSE+, PSD95+). DA neurons (DDC+, TH+, DAT+, VMAT2+, EN1+, LMX1A+and PITX3+)	Converted neurons can innervated in the nigrostriatal pathway and restore lost DA neurons and their axons within the nigrostriatal dopamine pathway	AAV-shPTB and PTB-ASOs induced DA neurons restored of striatal dopamine and reversed disease-relevant motor phenotypes.	Intracranial injection	Qian et al., 2020
Wild type mouse. NMDA-Induced retinal injury mouse model	CRISPER CasRx- Ptbp1	AAVs	Intact retinas. NMDA-Induced retinaas	Müller glia	Retinal ganglion cell (Brn3a+, Rbpms+)	Converted RGCs established central projections to dorsal lateral geniculate nucleus (dLGN) and superior colliculus (SC)	converted RGCs partially restored visual functions in a mouse model with drug-induced retinal injury	Intracranial injection	Zhou et al., 2020
Wild type mouse. 6-OHDA induced Parkinson’s disease (PD) mouse model	CRISPER CasRx- Ptbp1	AAVs	Intact striatum. PD striatum	Astrocytes	Substantia nigra pars compacta area-specific dopamine neuron (ALDH1A1+ and GIRK2+)	Induced neurons showing features of dopaminergic neurons in the striatum of PD model mice	Induced Neurons Alleviated Motor Dysfunctions in PD Mice	Intracranial injection	Zhou et al., 2020
Rat model of traumatic spinal cord injury	NeuroD1	AAVs	Dorsal horn of injured spinal cord	Reactive astrocytes	Spinal cord-specific glutamatergic neurons (Tlx3+)	NeuroD1-converted neurons can functionally mature and integrate into local spinal cord circuitry by displaying repetitive action potentials and spontaneous synaptic responses.	N/A	Intraspinal injection (peri-lesion region)	Puls et al., 2020
Rat model of traumatic spinal cord injury	NeuroD1 and Dlx2	AAVs	Dorsal horn of injured spinal cord	Reactive astrocytes	Spinal cord-specific GABAergic Neurons (Pax2+, Tlx3+)	N/A	N/A	Intraspinal injection (peri-lesion region)	Puls et al., 2020
Focal stroke model	NeuroD1	AAV9	Ischemic injured areas	Reactive astrocytes	Cortical pyramidal. neurons (Emx1+, Tbr1+, and Satb2+) and GABAergic neurons (parvalbumin+ and GABA+)	NeuroD1-mediated astrocyte-to-neuron conversion can trigger repetitive action potentials and form synaptic connections with other neurons in the injury sites after stroke	NeuroD1-treatment can reduce tissue loss after focal stroke. NeuroD1-treatment can rescue motor functional deficits following ischemic injury	Intracranial injection	Chen et al., 2020
Cuprizone induced demyelination model	Sox2	Lentivirus	Demyelinated corpus callosum	Reactive astrocytes	oligodendrocytes and OPCs (PLP+, PDGFRα+)	Induced-oligodendrocytes increased the level of myelination in Sox2-GFP treated animals	N/A	Intracranial injection	Farhangi et al., 2019
Intact mouse brain	NeuroD1	Lentivirus	Intact striatum	Microglia	Striatal projection neurons (βIII-tubulin+, Map2ab+, and DARPP32+)	NeuroD1-converted functionally integrated into brain circuits through synaptic connections with other neurons	N/A	Intracranial injection	Matsuda et al., 2019
Cuprizone induced demyelination model	Sox10	Lentivirus	Demyelinated corpus callosum	Reactive astrocytes	oligodendrocytes and OPCs (MBP+, PLP+, NG2+, Olig2+, PDGFRα+)	N/A	N/A	Intracranial injection	Mokhtarzadeh Khanghahi et al., 2018
6-OHDA lesions	Ascl1, Lmx1a, and Nurr1	AAV5	Intact and lesioned striatum	NG2 glia cells	DA neurons (TH+)	Induced neurons can integrate into existing brain circuitry and have properties of fast-spiking, parvalbumin-containing interneurons	N/A	Intracranial injection	Pereira et al., 2017
Intact mouse brain	NeuroD1	AAV9	Intact cortex and striatum	Resting astrocytes	Neurons (DCX+, NeuN+)	N/A	N/A	Jugular vein injection	Brulet et al., 2017
Intact mouse brain	Ascl1, Lmx1a, and Nurr2	AAV	Intact striatum	NG2 glia cells	GABAergic and glutamatergic neurons (NeuN+, MAP2+, vGlut1+, GAD65/67+)	Induced neurons showed functional electrophysiological properties and integrated into local circuitry	N/A	Intracranial injection	Torper et al., 2015
Mouse model of traumatic spinal cord injury	SOX2	Lentivirus	Injured spinal cord	Resting astrocytes	GABAergic neurons (GABA+, GAD65+)	N/A	N/A	Intraspinal injection (peri-lesion region)	Su et al., 2014
Mouse model of stab brain injury and Alzheimer’s disease	NeuroD1	Retrovirus	Injured cerebral cortex	Reactive astrocytes and NG2 glia cells	Glutamatergic neurons (vGluT1+) and GABAergic neurons (Tuj1+)	NeuroD1-converted neurons showing spontaneous and evoked synaptic responses	N/A	Intracranial injection	Guo et al., 2014
Intact rat brain	Ascl1, Brn2a, and Myt1l	Lentivirus	Intact striatum	Resting astrocytes	Neurons (NeuN+)	N/A	N/A	Intracranial injection	Torper et al., 2013

*N/A, not available.

## Potential to Cure for Alzheimer’s Disease Using *in vivo* Reprogramming Therapy

It has been proposed that glial cells play a critical role in neuritic plaques and NFTs in the pathological process of AD. Neuroglia cells are a fundamental element of the brain as they provide neurons with essential nutritive environments to maintain their normal function (Liu et al., 2021). Under the condition of injuries or diseases, they have the ability to repair impairments and promote neurogenesis in the early stages. However, this positive effect also has negative aspects. For example, reactive astrocytes, activated microglia, and nerve/glial antigen 2 (NG2) glia appear and participate in glial scar establishment. They formulate a hostile environment that blocks the reconstitution of neuron networks. During AD development, astrocytes appear to increase and become activated around the areas of amyloid accumulation, and together with activated microglia, initiate an immune cascade. The harmful effects increase with the time of degeneration (Martins et al., 2001; Toledano et al., 2016).

Studies have demonstrated that more functionally induced neurons and oligodendrocytes can be obtained by *in vivo* reprogramming (Farhangi et al., 2019; Wu et al., 2020; Zhou et al., 2020). *In vivo* reprogramming has been attempted in neurological diseases, such as Parkinson’s disease (Theodorou et al., 2015; Niu et al., 2018) and Huntington’s disease (Wu et al., 2020; Yu et al., 2021). Experiments have shown that reactive glial cells can be converted into functional neurons using a single TF NeuroD1 fate *in vivo* in brain injury and AD models (Guo et al., 2014). Resident astrocytes can be converted to doublecortin (DCX)-positive neuroblasts by a single transcription factor, SOX2, in the injured adult spinal cord. Importantly, these induced neuroblasts can mature into synapse-forming neurons *in vivo* (Su et al., 2014). *In vivo* reprogramming using Yamanaka factors (Oct4, Sox2, Klf4, and c-Myc) ameliorates aging features in dentate gyrus cells and improves memory in mice (Rodriguez-Matellan et al., 2020). The results suggest that *in vivo* reprogramming may be an effective strategy for improving CNS aging-related and neurodegenerative diseases, including AD.

Although previous research demonstrated that iPSCs grafted to AD mouse model reduced plaque deposition and improved cognitive dysfunction (Cha et al., 2017), and that reactive glial cells in the cortex of AD model mice can be directly reprogrammed into functional neurons *in vivo* using retroviral expression of a single neural transcription factor, NeuroD1 (Guo et al., 2014), only a few studies have reported *in vivo* reprogramming in AD animal models. Guo et al. (2014) injected NeuroD1-GFP retrovirus (CAG promoter) into the cortex of 5xFAD mice. The 5xFAD mouse model, used as a model of EOAD, expresses human APP and PSEN1 transgenes with a total of five AD-linked mutations: the Swedish (K670N/M671L), Florida (I716V), and London (V717I) mutations in APP, and the M146L and L286V mutations in PSEN1. The results showed that the reactive astrocytes in the AD model mouse brain could be reprogrammed into neurons, and that these newly reprogrammed neurons are functionally connected with surrounding neurons, which was confirmed by robust synaptic events in NeuroD1-converted neurons recorded in cortical slices (Guo et al., 2014). In the experiment conducted by Ghasemi-Kasman et al. (2018), microRNA-302/367 (miR-302/367)-expressing lentiviral particles were injected into the left hippocampal dentate gyrus of a mouse model of AD induced by intracerebroventricular injection (icv) of streptozotocin (STZ). Mouse model generated by administration of STZ via icv display numerous LOAD abnormalities. Brain insulin resistance, decreased brain glucose metabolism, cholinergic deficits, tau and Aβ accumulation, oxidative stress, gliosis, and learning and memory deficits have been reported in icv-STZ mouse model (Chen et al., 2013). The results demonstrated that miR-302/367 converted reactive astrocytes into neurons in AD mouse brains, and the induced neurons could fire repetitive action potentials like endogenous neurons confirmed by patch-clamp recordings. *In vivo* reprogramming with miR-302/367 significantly improved spontaneous alternation and spatial memory (Ghasemi-Kasman et al., 2018). Chronic inflammation is one of the pathological features of AD brains. *In vivo* reprogramming can convert glial cells into neurons and improve AD symptoms. Whether its role is related to the regulation of AD brain neuroinflammation has not been reported. Since *in vivo* reprogramming converts glial cells into neurons, resulting in a decrease in glial cells, it is reasonable to speculate that it has a role in reducing neuroinflammation and warrants further investigation.

*In vivo* reprogramming brings a new perspective to the treatment of AD, which possesses many advantages over *in vitro* reprogramming. First, compared to iPSCs and direct *in vitro* reprogramming, the working time in *in vivo* methods is significantly shortened, although a period of *in vivo* reprogramming is required. Second, the number of adverse reactions of resident aberrant glial cells decreases, instead, the number of functional neurons increases. Third, there is no pluripotent transition state process to go through (Srivastava and DeWitt, 2016). However, a fact that cannot be ignored is how to optimize the delivery media and pathways for TFs, microRNAs, and small molecules. For example, retrovirus and lentivirus vectors are the most commonly used vectors in basic research, and are delivered straight by injection into the brain, however, this method of delivery is a major obstacle to reprogramming *in vivo*. Other concern includes that, even if the functional neurons are induced, identified, and confirmed to take part in the local neuron network reconstruction, whether these changes can improve neurofunction remains unclear (Guo et al., 2014). Because of the extensive pathology of AD, it is also questionable whether local injection of acting factors can improve clinic symptoms of AD. It’s worth knowing whether multiple injections and multiple doses are better at reversing cognitive impairment. In addition, their optimal dose and frequency, as well as their carcinogenicity or inflammatory effects, remain unclear and need to be clarified.

Interestingly, existing study has shown that adeno-associated virus (AAV) vector serotypes 7, 8, 9, and Rh10 have brain-targeted properties (Cearley and Wolfe, 2006). Administration of adeno-associated virus (AAV) 9 intravenously can bypass blood brain barrier (BBB) and efficiently targets CNS cells, resulting in extensive transduction of dorsal root ganglia and motor neurons throughout the spinal cord and widespread transduction of neurons throughout the brain (Foust et al., 2009). In another *in vivo* cellular reprogramming research, adeno-associated virus 9 (AAV9) was used to deliver NEUROD1 to astrocytes through an intravascular route, and the results demonstrated that NeuroD1 integrated into the AAV9 vector, could convert non-reactive astrocytes into neurons in the striatum (Brulet et al., 2017). These approaches may enable the development of gene therapies for wide-ranging degenerative diseases, such as AD (Mingozzi and High, 2011).

## Perspective

Cellular reprogramming is a milestone technique in the development of AD therapy. Compared to iPSCs, *in vivo* reprogramming has unparalleled advantages, such as being more stable and able to mobilize resident normal/abnormal non-neuronal cells and local complex intra-cephalic microenvironments to participate in tissue repair. The *in vivo* reprogramming method can sufficiently produce functional neurons *in situ* and has great potential as a novel treatment for AD. However, there are still many issues to be resolved in its application in AD treatment. For examples, it is well known that astrocytes, microglia, and NG2 cells, even fibroblasts, can be directly reprogrammed into neurons. However, the selection of cell types to be reprogrammed, and their advantages and disadvantages, remain to be determined. The selected vectors, transcription factors, microRNAs or small molecules, or the optimal combination still require further study. In addition, the intrinsic mechanisms of the establishment and integration of neuronal networks remain under investigation. On the other hand, since Aβ plaques and hyperphosphorylated tau are neurotoxic, and excessive neuroinflammation can lead to nerve damage and neuronal death, whether the pathological environment of these ADs affects the effects of reprogramming and its effects on induced neurons should be addressed. However, this question has not been studied, expects future research. More importantly, the assessment of neurological functions should be used as an important indicator to evaluate the effects of different methods on AD treatment.

## Author Contributions

CZ conceived and wrote the manuscript. WN wrote part of the manuscript. FH critically revised and edited the manuscript. SD, TZ, and PS revised the manuscript. All authors contributed to the article and approved the submitted version.

## Conflict of Interest

The authors declare that the research was conducted in the absence of any commercial or financial relationships that could be construed as a potential conflict of interest.

## Publisher’s Note

All claims expressed in this article are solely those of the authors and do not necessarily represent those of their affiliated organizations, or those of the publisher, the editors and the reviewers. Any product that may be evaluated in this article, or claim that may be made by its manufacturer, is not guaranteed or endorsed by the publisher.
